# Challenges and opportunities of human iPSC-derived NK as “Off-the-shelf” cellular therapies

**DOI:** 10.1186/s13046-025-03558-6

**Published:** 2025-12-29

**Authors:** Nicola Romanini, Ratchapong Netsrithong, Maria Themeli, Marcella Tazzari

**Affiliations:** 1 Advanced Cellular Therapies and Rare Tumors Unit, IRCCS Istituto Romagnolo per lo Studio dei Tumori (IRST) “Dino Amadori”, Meldola, Italy; 2 Department of Medicine, Surgery and Neurosciences, University of Siena, Siena, 53100 Italy; 3 Department of Hematology, Amsterdam University Medical Center (UMC), Vrije Universiteit Amsterdam, Amsterdam, Netherlands; 4 Cancer Biology and Immunology, Cancer Center Amsterdam, Amsterdam, Netherlands

**Keywords:** Human induced pluripotent stem cell, Natural killer, Genome editing, Chimeric antigen receptor, Cell therapy, Clinical trial

## Abstract

The field of human induced pluripotent stem cell (hiPSC)-derived cell therapies is rapidly advancing, offering a promising “off-the-shelf” approach for treating both solid and hematologic malignancies. Among these, hiPSC-derived Natural Killer (NK) cell therapies have gained significant traction, with several currently in clinical trials and development. NK cell-based immunotherapy has emerged as a safe and effective strategy for patients with advanced leukemia, and ongoing research is focused on optimizing its accessibility, scalability, and efficacy. A key advantage of hiPSC-derived NK cells is their genetic susceptibility, allowing for targeted enhancements in fitness, metabolism, specificity, and cytotoxicity. This overcomes the donor-dependent variability that limits autologous and allogeneic NK cell therapies, which often struggle with expansion and functional consistency. Despite their promise, hiPSC-derived NK cells present unique manufacturing challenges, requiring precise optimization to ensure reproducibility, safety, and clinical-grade scalability. In this review, we will explore what we believe to be the most impactful genetic engineering strategies to enhance hiPSC-derived NK cell function. Additionally, we will also discuss the major hurdles challenging widespread clinical adoption, including licensing constraints, production yield, regulatory ambiguities, and the complexities of multi-step genetic engineering and safety validation. Finally, we will outline the emerging therapeutic pipelines from leading biotech companies, providing a valuable and up-to-date overview of the future landscape of hiPSC-derived NK cell therapy.

## Introduction

Advanced Therapy Medicinal Products (ATMPs), particularly cellular therapies, represent a promising frontier in the treatment of various diseases, with a strong focus on cancer and immune system disorders. Unlike autologous cell products, which are derived from a patient’s own cells, allogeneic cell therapies are designed as “off-the-shelf” solutions, offering standardized and scalable therapeutic options [1]. Allogeneic cell therapies could overcome two major issues of autologous Adoptive Cell Therapies (ACT): the occurring failure of autologous immune cell expansion from heavily pretreated patients, and the lack of manufacturing time in patients experiencing rapid disease progression [2, 3]. Moreover, even in the allogeneic setting, the individual healthy donor source still introduces donor-dependent variabilities potentially affecting responsiveness to genomic editing, cell replication yield, and cytotoxicity [4]. Among these, allogeneic NK cells, either in advanced pre-clinical studies or already applied in clinical trials, are exponentially increasing [5–7]. Particularly, the generation of Good Manufacturing Practices compliant (GMPc) NK cells has so far been conducted starting from a large variety of allogeneic cell sources [8–10]. Peripheral Blood (PB) is one of the most widely used methods, and cells obtained by this route carry mainly a mature phenotype, with greater cytotoxicity, but a shorter half-life and reduced replicative capacity (CD57^+^ and CD56^dim^ population). Secondly, Cord Blood (CB) represents a richer source of NK cells with a more naive phenotype compared to PB (CD57^low/dim^), and also displays different receptor characteristics, such as reduced expression of CD62L needed for lymph node homing. Moreover, CB is also an excellent source for CD34^+^ progenitors. A higher starting amount of CD34^+^ immature cells leads to several advantages, such as a greater fold of expansion and a less stringent time frame for any genetic modifications [11]. Conversely, while Bone Marrow (BM) serves as a valuable source of CD34^+^ progenitors, its procurement is more complex and therefore less frequently utilized. In general, CD34^+^ cells, although requiring extended culture time and careful handling during differentiation, exhibit greater susceptibility to genetic modifications and can be maintained in culture for longer periods, offering increased flexibility for downstream applications [12]. A fundamentally different approach involves the use of clonal NK cell lines, such as NK-92, a tumor-derived, immortalized cell line. NK-92 cells are highly amenable to genetic engineering, exhibit low sensitivity to cryopreservation, and are widely used in research due to their proliferative potential and well-characterized cytotoxic profile. Of note, they naturally lack endogenous CD16 (unless genetically modified) and do not express killer-cell immunoglobulin-like receptors (KIRs) [13]. Most critically, their tumor-derived origin necessitates irradiation before infusion to prevent uncontrolled proliferation, thereby limiting their in vivo persistence. Recently, the use of hiPSC has emerged as an alternative, unlimited source of allogeneic NK cells. Notably, hiPSCs are highly amenable to genetic manipulation and offer the greatest potential for generating large, reproducible cell populations, aligning with the “off-the-shelf” concept. Moreover, NK cells derived from hiPSCs are typically immature but can be expanded and matured into more cytotoxic phenotypes. To enhance their anti-tumor potential, genome editing strategies are often required to boost cytotoxicity while ensuring sufficient expansion and persistence. Indeed, one of the major limitations of adoptive NK cell therapies in solid tumors is the limited persistence of transferred cells. Unlike T cells, which can give rise to long-lived memory populations over the host’s lifespan, NK cells are characterized by a relatively rapid turnover and short lifespan, with only limited capacity for prolonged survival and sustained functional activity in circulation [14]. Table 1 provides an overview of the key strengths and limitations associated with each NK cell platform. In this review, we discuss the potential advantages and key challenges of translating hiPSC-derived NK cells into clinical applications.

**Table 1:** Comparative features of NK cell platforms

NK Platform	Key Strengths	Key Limitations	Manufacturing	Clinical/Preclinical
**PB-NK (Peripheral Blood)**	- Mature phenotype with high cytotoxicity and ADCC capability via CD16 - Readily available via apheresis from healthy donors	- Limited cell yield per donor - Reduced function post-thaw - Short lifespan after infusion - Difficult to standardize: variability in phenotype/function between donors - Requires donor matching or allogeneic protocols for off-the-shelf use - Moderate genetic modificability	~ 2–3 weeks	Clinical Trials
**CB-NK (Cord Blood NK)**	- Off-the-shelf availability via cord blood banks - High proliferative potential - Low risk of GVHD	- Immature phenotype with low KIR/CD16 expression - Difficult to standardize: variability in phenotype/function between donors - Function may decline post-thaw	~ 2–3 weeks	Clinical Trials
**HSPC-CB-NK (Cord Blood–derived Hematopoietic Stem/Progenitor Cell NK)**	- Uniform product from defined HSPC source - Amenable to feeder-based or feeder-free expansion and genetic engineering - Potential for large-scale GMP manufacturing - Low risk of GVHD	- Requires complex differentiation protocols and long culture times - Maturation to fully functional NK phenotype can be incomplete leading to low CD16, limiting ADCC without engineering	~ 4–5 weeks	Preclinical/early trials
**iPSC-NK (induced Pluripotent Stem Cell-derived NK)**	- Virtually unlimited, uniform “off-the-shelf” source - Highly amenable to multiplex gene editing (CAR, cytokine support, checkpoint modulation) - Flexible differentiation strategies to bias toward cytotoxic or memory-like features - Homogeneous cell products - Minimal immune rejection	- Long differentiation time and phenotype may remain immature (low KIR/CD16) without optimized maturation steps - Complex and expensive manufacturing protocols, as well as variability between manufacturing sites - Higher regulatory complexity as a novel cell source and need of sophisticated infrastructures	~ 5–9 weeks	Preclinical/early trials
**NK-92 Cell Line**	- Robust in vitro expansion - Homogeneous, well-characterized population - easy to engineer (CAR, cytokine genes) - No donor variability	- Requires irradiation prior to infusion (non-proliferative in vivo), limiting persistence - Tumor-derived origin raises safety concerns - Lacks CD16, limiting ADCC without engineering	~ 1–2 weeks	Clinical Trials

## hiPSC platform

**Fig. 1:**
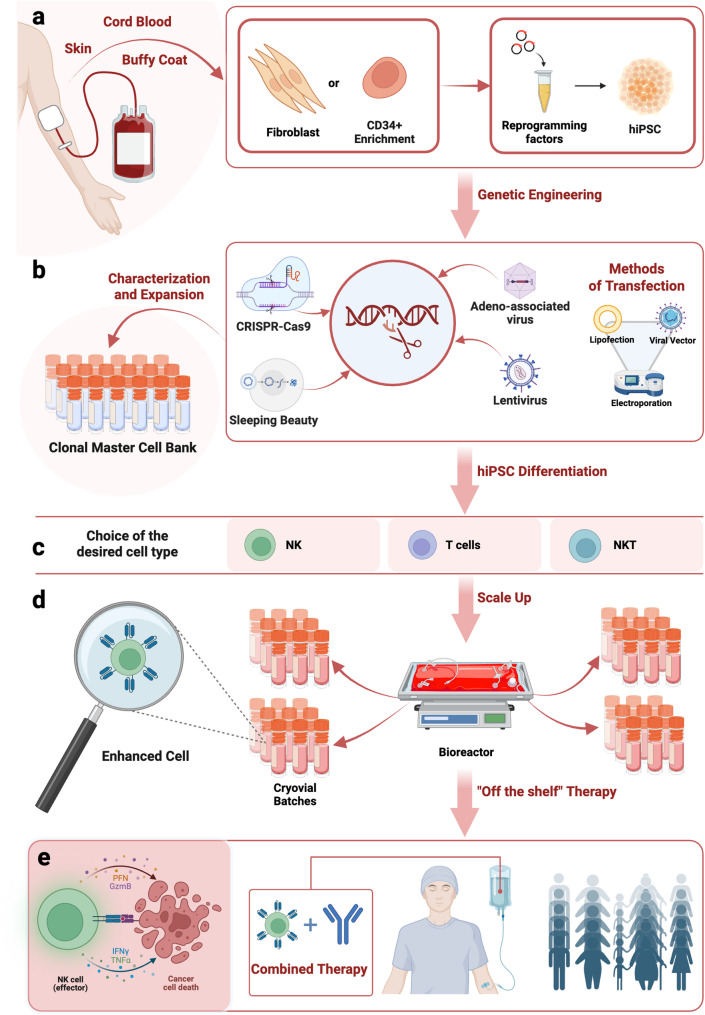
Overview of the hiPSC-based immunotherapy platform. **a** Representative examples of available hiPSC resources. **b** Overview of transfection methods and genome editing tools used to introduce therapeutic modifications, followed by clonal selection and expansion to establish a genetically defined MCB. **c** Directed differentiation of hiPSCs into immune effector cell types for use in adoptive cell therapies. The platform allows dynamic adaptation of cell fate according to emerging therapeutic needs. **d** Transition from lab-scale protocols to industrial-scale production using bioreactors, enabling the generation of clinically relevant, time-sustainable cell therapy batches. **e** Clinical infusion of hiPSC-derived products, potentially in combination with adjuvant agents. The therapeutic strategy is tailored based on the genetic engineering approach and cell lineage; for instance, co-administration of opsonizing antibodies is often recommended in NK cell-based therapies

The hiPSC platform is centered on the establishment of well-characterized hiPSC Master Cell Banks (MCB) through a series of critical steps. Primary cell sources include fibroblasts, harvested from low-UV-exposed skin areas of a young donor, or CD34^+^ progenitor cells (Fig. 1a). Reprogramming is then performed to induce pluripotency. Initially, hiPSCs were obtained by a viral transduction of a cocktail of transcription factors devised by Takahashi and Yamanaka, consisting of Krüppel-like factor 4 (Klf4), Octamer-binding transcription factor 3/4 (Oct3/4), SRY (sex determining region Y)-box 2 (Sox2), and Cellular-Myelocytomatosis (c-Myc) (OSKM) [15–17]. Subsequently, more efficient and durable systems were developed to express these transcription factors, which are divided into integrative or non-integrative and viral or non-viral transfer systems. For clinical translatability, non-integrating reprogramming methods such as miRNAs, mRNAs, plasmids, or Sendai viruses are preferred over lentiviruses or retroviruses to avoid regulatory hurdles related to insertional mutagenesis and enhance safety [18, 19]. Then, multi-step genetic modifications generate batches for different therapeutic targets (Fig. 1b). Each genetic engineering platform presents specific advantages and limitations: viral vectors ensure high transduction efficiency but carry a risk of insertional mutagenesis, while gene-editing technologies such as CRISPR-Cas9 offer greater precision, thereby enhancing safety and facilitating compliance with GMP standards for clinical translation. A critical aspect of the CRISPR platform optimization is the improvement of gene-editing efficiency while maintaining cell viability. Once the genetic modifications are successfully introduced, validated hiPSC clones are isolated, expanded, and selected to derive the final clonal master hiPSC line (Fig. 1c), finally resulting in product homogeneity and consistency [20]. The differentiated cells will retain the introduced modifications, enabling the scalable production of “off-the-shelf” cell populations for multiple patient doses. In the field of cell therapies, the most promising alternatives include the development of T lymphocytes, NKT cells, and NK cells [21]. Unlike Chimeric Antigen Receptor (CAR)-T cell therapies, NK cells are demonstrating a better safety profile in clinical trials. They do not produce severe toxicity effects such as graft-versus-host disease (GvHD) or cytokine release syndrome (CRS), proving to be one of the best resources for cancer therapy [22–25]. In addition, NK cells are able to kill cancer cells in an antigen-independent manner and do not require Human Leukocyte Antigens (HLA) matching, unlike T lymphocytes. These features make it a viable option for “off-the-shelf therapy”. Once the cells have been differentiated from the clonal master hiPSC line, scale-up is carried out in bioreactors to expand cells and reduce manpower. Several bioreactors are available in the market including wave bioreactors such as the Cytiva Xuri™ Cell Expansion System, Sartorius Biostat^®^ RM, and Thermo Scientific HyPerforma™ Rocker Bioreactor, cell processing platforms such as the Miltenyi CliniMACS Prodigy^®^, Lonza Cocoon^®^ Cell Therapy Manufacturing Platform, and static bioreactors such as Wilson Wolf G-Rex^®^ (Fig. 1d). Large-scale production of cell therapies requires the use of advanced bioprocessing techniques, which can be expensive and complex [26, 27]. Cell batches produced in bioreactors can be cryopreserved in liquid nitrogen until needed for clinical use. Prior to infusion, patients typically undergo a preconditioning regimen, such as chemotherapy or immunosuppressive treatment, to modulate the immune environment and enhance engraftment, persistence, and efficacy of the adoptively transferred cells. NK cell therapy can be combined with immune checkpoint inhibitors to overcome tumor immune evasion and/or with monoclonal antibodies targeting tumor-specific antigens, thereby engaging CD16-mediated Antibody-Dependent Cell-mediated Cytotoxicity (ADCC) to enhance antitumor efficacy (Fig. 1e) [28].

## Insights from transcriptomic, epigenetic profiling, and comparative analyses

Phenotypic analysis, primarily based on the evaluation of surface markers, continues to serve as a cornerstone of in-process control and release testing in cell therapy. However, this approach alone is insufficient to fully capture the complexity and heterogeneity inherent to therapeutic cell populations. In contrast, transcriptomic profiling, both at bulk and single-cell resolution, has emerged as a powerful tool to uncover the identity, diversity, and functional states of immune cells, including NK cells [29–31]. Nonetheless, a deeper understanding of NK cell ontogeny is essential to refine protocols for the ex vivo generation of therapeutic NK cells from CD34⁺ hematopoietic stem cells or hiPSCs. To this aim, Zhang and colleagues performed single-cell RNA sequencing across the differentiation trajectory from hiPSCs to NK cells. By comparing five hiPSC clones with varying efficiencies of hematopoietic lineage commitment (CD34⁺), they found that the best-performing clones exhibited increased expression of transcription factors critical for NK cell differentiation, such as EOMES. These findings suggest that NK cell production could be improved even prior to differentiation by optimizing the hematopoietic priming status of hiPSCs [32]. Traditionally, NK cell development was described by a linear model, whereby NK cells arise from common lymphoid progenitors (CLPs). However, a branched model has been proposed, suggesting that lymphoid-primed multipotent progenitors (LMPPs) give rise to both CLPs and common myeloid progenitors (CMPs), each capable of differentiating into NK cells [33]. Van Vliet and colleagues combined bulk and single-cell RNA sequencing at two distinct stages of NK differentiation to investigate the molecular heterogeneity of 10 umbilical cord blood-derived NK cell batches previously categorized as either “excellent” or “good” killers based on their in vitro cytotoxicity against solid and hematological cancer lines. Notably, “excellent” donors displayed an enrichment of cytotoxicity pathways and a depletion of myeloid traits, associated with the early emergence of effector-like NK populations [31]. Goldenson et al. attempted to compare the transcriptional landscapes of hiPSC-derived NK cells and CB-derived NK cells [34]; however, no major differences were observed, likely due to the homogenizing effect of post-expansion using irradiated artificial antigen-presenting cells (aAPCs), namely K562 expressing IL-21 and 4-1BBL. Indeed, comparative transcriptomic analyses across different hiPSC-derived NK differentiation protocols have further highlighted how not only the NK cell source but also the specific differentiation strategy significantly shape the final NK phenotype and function. Huyghe and colleagues conducted a direct comparison of NK cells generated using two distinct differentiation protocols from the same hiPSC line: (i) a short-term, clinically compatible feeder-free protocol, and (ii) a stromal-based OP9-DLL4 protocol representing the definitive hematopoietic stage. Despite the identical starting material, the resulting NK cell populations exhibited marked differences in phenotype, transcriptomic profiles, and functional capacity. Notably, NK cells derived via the OP9-DLL4 method showed a more mature and activated phenotype, with higher surface expression of activating receptors such as NKp80, NKp30, NKp46, and DNAM-1. At the transcriptomic level, they also exhibited increased expression of key transcription factors associated with NK maturation and function, including ZEB2, STAT1, FOSB, and JUN, further confirming their advanced differentiation status. Functionally, OP9-DLL4-derived NK cells outperformed their feeder-free counterparts in vitro. When co-cultured with K562 target cells, they demonstrated superior degranulation capacity, higher short-term cytotoxic activity, and greater IFN-γ production. These insights underscore the importance of tailoring differentiation conditions to achieve the desired NK cell functional outputs. Furthermore, recent studies on memory-like NK cells offer a valuable framework to evaluate the potential of hiPSC-derived NK cells to acquire long-lived, enhanced functional states [25, 35–37]. Incorporating memory-like programming into hiPSC platforms, mimicking the effects of short-term cytokine preactivation with IL-12, IL-15, and IL-18, could be a promising strategy to boost the persistence and antitumor efficacy of NK cell therapies [25, 37]. This is in line with seminal studies in mice, humans, and non-human primates have demonstrated that NK cells, despite being part of the innate immune system, can acquire “memory-like” or “adaptive” features, characterized by prolonged survival and enhanced recall responses [38–42]. These properties were initially observed in viral infection models and, more recently, have been linked to improved antitumor effects and prolonged persistence in certain clinical settings, such as myeloid leukemia [43–46]. Notably, Rückert and colleagues demonstrated, in the context of human cytomegalovirus (HCMV) infection, that clonal inheritance of chromatin accessibility defines the epigenetic memory repertoire of NK cells. This finding underscores that NK cells can adopt adaptive-like properties through non-genetic, heritable epigenetic mechanisms, independent of antigen-receptor diversification. However, while these observations provide a strong rationale for exploring memory-like NK cells in cancer immunotherapy, direct evidence from tumor rechallenge models remains limited, and their clinical relevance in cancer has yet to be firmly established. It also remains unclear whether NK cells are capable of mounting true antigen-specific recall responses upon tumor rechallenge. It must also be considered that the power of non-genetic inheritance mechanisms in influencing ATMP fitness can stem from the epigenetic makeup of the somatic cell of origin, significantly impacting the differentiation trajectory and transcriptional program of hiPSC-derived NK cells. This highlights that hiPSC source selection remains a non-trivial consideration in the context of therapeutic manufacturing [47]. Altogether, these transcriptomic and comparative analyses not only deepen our mechanistic understanding of NK cell biology but also reveal that the therapeutic performance of NK cell products is multifactorial. Both the intrinsic hematopoietic priming status of hiPSCs and the specific cues provided during in vitro differentiation critically steer the developmental trajectory of NK progenitors. As stressed by others, reliance solely on phenotypic markers should be approached with caution, given the dynamic changes induced during ex vivo culture conditions [31]. Instead, integrating high-resolution transcriptomic analyses provides an unprecedented window into the mechanisms regulating NK cell development and function, enabling a functional interpretation of donor variability and supporting the identification or engineering of superior NK cell effectors, critical steps toward the rational design of next-generation hiPSC-based immunotherapies.

Importantly, these insights should be integrated with emerging evidence highlighting the role of epigenetic regulation, including the residual memory of the somatic cell of origin, in shaping transcriptional programs and influencing the differentiation potential of hiPSC-derived NK cells. Epigenetic imprinting thus represents an additional, often overlooked, layer of complexity in the manufacturing of advanced NK cell therapies.

## Genetic engineering strategies to enhance NK cell function

**Fig. 2:**
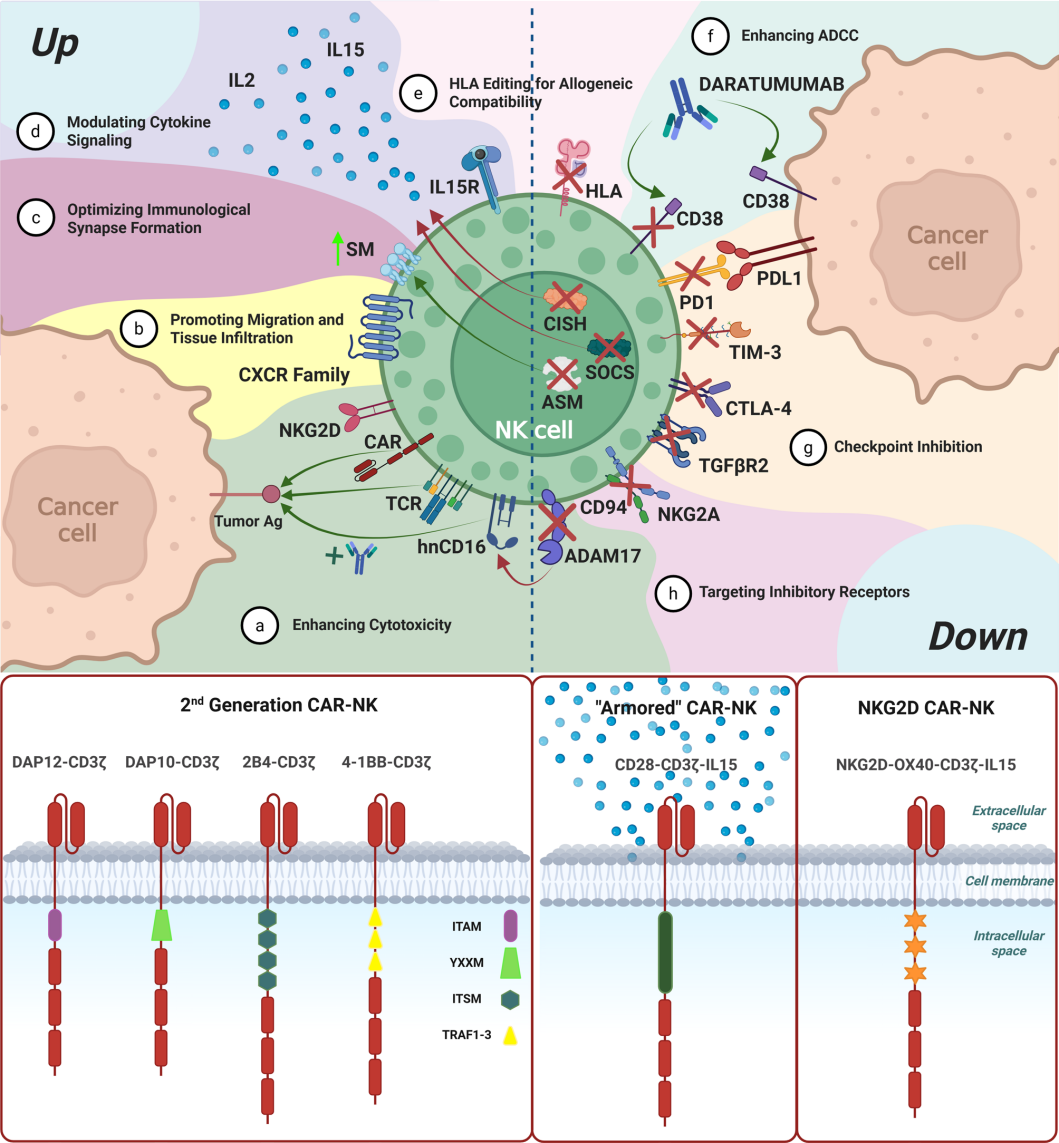
Overview of genetic engineering strategies to enhance NK cell therapeutic function. **a** Enhancing cytotoxicity: engineering approaches aim to increase NK cell tumor-killing ability: second-generation CAR-NK cells, modeled after CAR-T constructs but tailored to NK-specific signaling pathways; armored CAR-NK cells engineered to secrete immunostimulatory cytokines; and NKG2D-based CARs, which amplify endogenous activating signals. **b** Promoting migration and tissue infiltration: expression of selected chemokine receptors and adhesion molecules facilitates NK cell homing, infiltration, and retention into tumor tissues. **c** Optimizing immunological synapse formation: structural modifications, such as knockdown of the catabolic enzyme ASM, improve membrane dynamics and synapse formation, enabling effective cytotoxic engagement even in metabolically hostile TMEs. **d** Modulating cytokine signaling: overexpression of cytokines, such as IL-15 and IL-2 or removal of the negative regulator CISH, enhances NK cell proliferation, persistence, and effector function, supporting robust anti-tumor immunity. **e** HLA editing for allogeneic compatibility: deletion of HLA molecules reduce the risk of alloreactivity and immune rejection. **f** Enhancing ADCC: co-administration of tumor-targeting antibodies (e.g., Daratumumab for CD38) can be paired with CD38 knockout in NK cells to avoid fratricide, while ADAM17 deletion or hnCD16 expression enhances sustained CD16-mediated killing. **g** Checkpoint inhibition: genetic disruption of inhibitory immune checkpoints (e.g., TIGIT, PD-1, CTLA-4, TIM-3) enhances NK cell activation and reverses tumor-induced exhaustion, promoting durable cytotoxic responses. **h** Targeting inhibitory receptors: deletion of NK-specific inhibitory receptors such as NKG2A or KIRs shifts the activation threshold, improving tumor cell recognition and cytotoxic activity

NKs possess a complex repertoire of activating and inhibitory receptors that enables them to distinguish effectively between healthy and pathological cells, as extensively described by Wang et al. [48]. Harnessing and enhancing NK cell function is a critical objective in the development of effective antitumor immunotherapies. In the following section, we summarize what we consider the most promising gene-editing strategies currently under investigation, both gain-of-function and loss-of-function approaches, that aim to potentiate NK cell antitumor activity. (Fig. 2).

### Enhancing Cytotoxicity and Target Recognition

#### Genetic introduction of CAR or T cell receptor (TCR)

Among the most relevant modifications is the introduction of a CAR, which enhances cytotoxicity by enabling antigen-specific targeting [49]. Antigen recognition typically relies on a single-chain variable fragment (scFv) of a selected antibody (Fig. 2a), though CARs utilizing extracellular domains of native cellular receptors have also been developed to exploit natural receptor-ligand interactions [50]. Originally designed for T cells, they have been adapted to NK cells with NK cell-optimized constructs replacing CD3ζ with NK-specific signaling domains such as DNAX-activating protein 12 (DAP12) and DAP10. DAP12 contains immune-tyrosine activation motifs (ITAMs) that trigger pro-inflammatory cytokine release and cytotoxic granule exocytosis upon phosphorylation, while DAP10, which lacks ITAMs, signals via the NKG2D-DAP10 axis to promote strong NK activation. Second-generation CARs for NK cells often combine NK-specific domains with CD3ζ (e.g., DAP12-CD3ζ; DAP10-CD3ζ; 2B4-CD3ζ and 4-1BB-CD3ζ), showing superior activity to early designs. Third-generation CARs include two or more costimulatory domains (2B4, CD28, DAP10, DAP12, 4-1BB), improving NK cell survival, proliferation, and cytotoxicity [24]. “Armored” CARs, such as CD28-CD3ζ-IL-15, further enhance cytokine production and sustain NK cell functionality in the tumor microenvironment (TME) [51–54]. Beyond CARs, TCR engineering can confer specificity against intracellular antigens presented on MHC class I, expanding the range of targetable tumor-associated antigens beyond surface proteins. However, TCR-based therapies face challenges such as tumor-mediated MHC-I downregulation and the requirement for HLA compatibility, which limits broad applicability [55, 56].

#### Upregulation of native activating receptors

To further enhance NK cell cytotoxicity, genetic engineering strategies can include the overexpression of native activating receptors (Fig. 2a). One notable example is NKG2D, a key receptor that triggers target cell lysis upon recognizing stress-induced ligands frequently expressed on tumor cells. This approach has been harnessed in the design of the synthetic receptor NKX101, which integrates NKG2D with co-stimulatory and cytokine domains (OX40-CD3ζ-IL-15), and has shown encouraging anti-leukemic efficacy in preclinical models [57]. Beyond NKG2D, other native death-inducing receptors, such as Fas (CD95) and TRAIL (CD253), can also be upregulated to potentiate NK cell-mediated apoptosis. ONK Therapeutics, for example, has developed a high-affinity variant of the TNF-related apoptosis-inducing ligand (TRAILv), which adds a secondary apoptotic mechanism that complements CAR-driven cytotoxicity [58].

### Optimizing trafficking and tumor homing

Effective homing of NK cells to tumor sites remains a key challenge in adoptive immunotherapy. Multiple studies have demonstrated that modulation of specific chemokine receptors can enhance tissue-specific trafficking. For instance, E. Levi and colleagues reported that upregulation of CXCR4 in NK cells significantly improved their homing to the BM [59]. Similarly, V. Kremer et al. showed that enforced expression of CXCR2 promoted NK cell infiltration into renal carcinoma lesions [60]. In ovarian cancer models, electroporation of NK cells with CXCR1 mRNA enhanced their migration to tumor sites and improved tumor control in vivo [61]. Additional targets such as CXCR7 and CD62L, typically absent or weakly expressed on NK cells, have also been implicated in improving migration, particularly to lymphoid tissues, when artificially upregulated. Moreover, Shannon et al. provided a comprehensive review of the integrins and adhesion molecules involved in NK cell trafficking across various tissue types. They could be exploited to improve tissue persistence and migration to the organ of interest [62], highlighting molecules that may be leveraged to improve tissue retention and organ-specific localization. Collectively, these findings emphasize the therapeutic potential of engineering NK cells to express specific homing receptors and adhesion molecules to enhance their recruitment and persistence within the tumor site [63] (Fig. 2b).

### Improving NK cell persistence and fitness

A major limitation of NK cell-based therapies is their relatively short persistence in vivo, which can compromise therapeutic efficacy. To overcome this, various strategies have been developed to enhance NK cell survival, proliferation, and resistance to senescence. One of the most promising approaches involves augmenting IL-15 signaling, which plays a critical role in NK cell homeostasis and function (Fig. 2d). Enhanced IL-15 stimulation has been shown to improve cytotoxicity, in vivo persistence, and differentiation. Some studies have introduced point mutations in the IL-15Rα binding domain, increasing receptor affinity for IL-15. Others have utilized affinity tags to stabilize the IL-15/IL-15Rα complex, thereby enhancing receptor clustering and intracellular signaling [64]. An alternative strategy has involved engineering NK cells to express a constitutively active IL-15 receptor, eliminating the need for ligand binding to trigger activation. This design has been successfully incorporated into therapeutic NK cell products, such as NKX019 and NKX101. Given the robust preclinical and early clinical data, genetic engineering to promote IL-15 signaling, or analogous strategies such as cytokine tethering, has become a recommended approach to enhance NK cell fitness and therapeutic efficacy [65, 66]. In parallel, IL-2 signaling pathways can also be leveraged to support NK cell proliferation, survival, and adhesion molecule expression. This can be achieved through genetic modifications, analogous to those used for IL-15, further broadening the toolkit for improving NK cell-based immunotherapy [67].

### Overcoming Tumor-Induced Immunosuppression

#### Metabolic adaptations for TME resistance

Enhancing the metabolic fitness of therapeutic NK cells is increasingly recognized as key to sustaining their function in the hypoxic, nutrient-poor, and immunosuppressive TME [68, 69]. Metabolic rewiring supports not only cytotoxicity but also trafficking, persistence, and responsiveness in hostile tumor niches. Dysregulated sphingomyelin (SM) metabolism, closely linked to serine availability, has been implicated in defective NK cell adhesion, migration, and cytotoxicity [70]. As shown by Xiaohu Zheng et al., NK cells isolated from intratumoral liver cancer regions exhibited defective membrane protrusions, limiting immune synapses formation and tumor infiltration. This was traced to overactive SM catabolism; knockdown of catabolic enzymes, such as acid sphingomyelinase (ASM) or neutral sphingomyelinases (NSMASE1, NSMASE2, and NSMASE3), restored membrane architecture and function (Fig. 2c). These findings highlight how metabolic and structural barriers in the TME synergize to suppress NK cell function, linking lipid metabolism and serine biosynthesis to trafficking, retention, and cytotoxicity. Targeting these pathways could improve NK-based immunotherapy. Beyond metabolic enzymes, modulating cytokine signaling pathways also offers a route to enhance NK cell fitness. Deletion of negative regulators such as CISH (Cytokine-inducible SH2-containing protein) and SOCS2 (Suppressor of cytokine signaling 2), members of the SOCS protein family, has shown promise [71–73]. CISH, a key intracellular checkpoint dampening IL-15 signaling, can be removed without affecting hiPSC maintenance, making it attractive for engineered NK platforms. Companies, such as ONK Therapeutics Limited and Shoreline Biosciences, have incorporated CISH knockout to generate metabolically optimized NK cells. Preclinical studies confirm that CISH removal improves IL-15 responsiveness and enhances NK cell function in tumors [74–76] (Fig. 2d).

#### Modulation of immunosuppressive signaling pathways and disruption of inhibitory receptors

Tumor-derived immunosuppressive signals markedly impair NK cell function, making their modulation a key strategy to enhance NK-based immunotherapies. One major pathway involves TGF-β (Transforming Growth Factor Beta), abundantly secreted by tumor cells, which suppresses NK activity via TGFβR2 (TGF-β receptor II); targeted deletion of TGFβR2 restore cytotoxic and improve NK cell performance in the TME [77–79] (Fig. 2g). Several immune checkpoint receptors, TIM-3, CTLA-4, TIGIT, PD-1 and others such as LAG3, also contribute to NK cell dysfunction. While well characterized in T cells, their role in NK cells is emerging, and their blockade or deletion can promote a more cytotoxic phenotype. For example, TIM-3 upregulation correlates with reduced NKG2D and TNF-α [80]; TIGIT induces NK exhaustion, and its inhibition boosts antitumor activity [81, 82]; CTLA-4 blockade shows preclinical efficacy [83], with knockout approaches under study [84]. Targeting the PD-1/PD-L1 axis can be achieved via PDCD1 (encoding PD-1) editing [85] or monoclonal antibodies such as Atezolizumab. Katharina et al. engineered NK cells with a PD-1–based chimeric switch receptor, converting inhibition into activation, enhancing degranulation, cytokine secretion, and cytotoxicity, upon PD-L1 encounter [86, 87]. In addition, disruption of NK-specific inhibitory receptors such as KIRs and NKG2A can further potentiate cytotoxicity and prevent exhaustion (Fig. 2h). NKG2A binds HLA-E, delivering inhibitory signals that impair immune synapse formation and NK-mediated killing [88, 89].

### Preventing fratricide and alloreactivity

To reduce the risk of alloreactivity and rejection in adoptive cell therapies, several studies have explored knocking out the beta-2-microglobulin (B2M) in NK cells to eliminate surface expression of the HLA class I complex (Fig. 2e). This strategy prevents recognition and destruction by the patient’s T cells, thereby increasing the compatibility of NK cells in allogeneic settings. To counteract fratricide, a phenomenon where NK cells attack each other due to the absence of self-HLA, researchers have co-engineered the expression of a single-chain HLA-E molecule. This modification provides an inhibitory signal through the “missing self” recognition pathway, effectively protecting NK cells from one another. Interestingly, the removal of HLA molecules not only mitigates alloreactivity but also renders NK cells less visible to the host’s immune system, allowing them to evade recognition by mismatched CD8⁺ and CD4⁺ T cells. This immune evasiveness makes them highly attractive candidates for “off-the-shelf” therapeutic applications. However, caution is warranted when combining this approach with NKG2A knockout, since NKG2A is one of the key inhibitory receptors that recognize HLA-E. Eliminating both HLA-E and its receptor may inadvertently remove critical self-tolerance checkpoints, thereby reintroducing the risk of NK cell fratricide [90, 91].

### Combination with therapeutic antibodies

The integration of therapeutic monoclonal antibodies with NK cell-based therapies is a well-established strategy to enhance tumor targeting through ADCC (Fig. 1e). A key innovation in this context is the engineering of NK cells to express high-affinity, non-cleavable CD16a (hnCD16), a modified version of the FcγRIIIa receptor that is resistant to shedding and exhibits stronger binding to therapeutic antibodies (Fig. 2a) [92, 93]. In ACT approaches, tumor-specific monoclonal antibodies can be co-administered to the patient to direct NK cell cytotoxicity toward defined targets. These targets may include tumor-associated antigens such as CD38, which is recognized by Daratumumab in multiple myeloma, or immune checkpoint ligands such as PD-L1, targeted by Atezolizumab (Figs. 1e and 2f). One challenge with CD38-targeted therapies is that NK cells themselves express CD38, making them vulnerable to fratricide when Daratumumab is used. To prevent this, CD38 gene knockout in NK cells has been employed to render them invisible to the antibody while preserving anti-tumor targeting. In addition to hnCD16 expression, another strategy to sustain ADCC functionality involves the deletion of *ADAM17*, a metalloprotease responsible for cleaving CD16 from the NK cell surface. Inhibiting or knocking out *ADAM17* prevents CD16 shedding, maintaining NK cell sensitivity to antibody engagement and enhancing cytotoxic signaling [85]. Co-administration of tumor-targeting antibodies has also been shown to increase NK cell adhesion, cytokine release, and overall anti-tumor function [94, 95]. Notably, the use of bispecific antibodies in clinical settings has demonstrated considerable promise. For instance, in the study by Nieto et al., a bispecific antibody simultaneously targeting CD16 and CD30 was pre-complexed onto ex vivo expanded NK cells. Upon infusion into patients with relapsed/refractory lymphoma, these pre-armed NK cells showed enhanced tumor recognition and potent cytotoxic activity [96].

Together, these combination strategies significantly broaden the therapeutic potential of NK cells and provide a flexible platform to target diverse tumor types with improved specificity and efficacy.

## Overview of biotechnology companies in the NK cell therapy field

The global hiPSC market is poised for significant growth, driven by rising incidences of chronic diseases and ongoing technological advancements. Valued at USD 1.92 billion in 2024, the market is projected to reach USD 5.60 billion by 2035, expanding at a compound annual growth rate (CAGR) of 10.23% between 2025 and 2035. North America currently dominates the market, followed by the Asia-Pacific region. The increasing prevalence of chronic conditions such as cardiovascular disease, cancer, and diabetes is accelerating demand for hiPSCs, which offer key advantages including personalized therapeutic potential and the absence of ethical concerns [97]. Currently, several biotechnology companies, such as Fate Therapeutics, Century Therapeutics, Hebecell, and Cytovia Therapeutics, are producing hiPSC-derived NK-based therapies (Table 2). Fate Therapeutics is a clinical-stage biopharmaceutical company that focuses on developing innovative cancer immunotherapies. One example of their hiPSC-derived NK cell-based products, FT596, was engineered to contain three gene modifications aimed at improving its efficacy against hematologic malignancies. The first gene modification involved the introduction of a non-cleavable variant of CD16 to enhance the ADCC of NK cells. The second modification was the introduction of a membrane-bound IL-15/IL15R fusion protein (IL-15RF) to enhance in vivo persistence, and the third modification was the addition of an anti-CD19 CAR optimized for NK cells for direct tumor targeting. These gene modifications were designed to address limitations of existing immunotherapies, such as loss of CD19 expression in relapsed patients treated with anti-CD19 CAR-T cell therapy. Preclinical studies have shown that FT596 cells are effective in eliminating both CD19^+^ and CD19^-^ lymphoma cells and have shown greater natural cytotoxicity, persistence, and targeted action on malignant cells compared to healthy B cells [98]. Many Fate’s Phase I clinical trials are currently underway to test the safety and preliminary efficacy (Table 2). Early results from these clinical trials show that these hiPSC-derived NK cell products demonstrate indications of efficacy, achieving in some cases a complete response, without dose-limiting toxicities or serious adverse events. More specifically, patients were treated with different doses of FT516, 3 × 10^7^ to 9 × 10^8^ cells per dose on days 1, 8, and 15, with IL-2 (6 million units) administered subcutaneously 2–4 h after each dose. There was a low incidence (2%) of cytokine release syndrome (CRS) and no neurotoxicity events. The most common adverse events grade 3 or worse were neutropenia (84%), thrombocytopenia (36%), and anaemia (27%). Anti-tumor activity was observed across all lymphoma types tested, and objective response was observed in 32 out of 55 patients (58%), with 24 (44%) of patients achieving a complete response. FT596 was used to treat 20 patients at different doses, either as monotherapy (regimen A) or in combination with Rituximab (regimen B). Similar to FT516, there was a low incidence of CRS and no neurotoxicity, while Grade ≥ 3 adverse events included neutropenia (78–88%), thrombocytopenia (39–49%), and anaemia (39–44%). Overall response rate was 54% with 37% of the patients achieving complete remission [99, 100]. In addition, Fate Therapeutics has several other ongoing Phase I clinical trials on hematological and solid cancers (Table 2) [101].

Century Therapeutics is conducting a Phase I study, called ELiPSE-1 (Table 2), to evaluate the efficacy, safety, tolerability, and pharmacokinetics of CNTY-101 in patients with relapsed or refractory CD19-positive B-cell lymphomas. CNTY-101 is a homogeneous hiPSC master cell monoclonal bank product where all cells present an anti-CD19 CAR, Allo-Evasion™ technology designed to overcome the three major host rejection pathways, and IL-15 support to improve cell function and persistence. In detail, the Allo-Evasion™ technology employs knock-out of β2M, to evade CD8^+^ T cells, knock-out of the Class II Major Histocompatibility Complex Transactivator (CIITA), in order to prevent recognition by CD4^+^ T cells, and knock-in of the HLA-E gene to increase HLA-E protein expression and prevent the killing of CNTY-101 cells by host NK cells. In addition, the product is engineered with an EGFR safety switch and thus, cells can be quickly eliminated, if needed, by the administration of Cetuximab (anti-EGFR), approved by the U.S. Food and Drug Administration (FDA) for certain tumor types. Cytovia Therapeutics has several CAR-NK products in the pre-clinical phase of development, such as CYT503 for hepatocellular carcinoma, CYT538 for multiple myeloma, and CYT501 for glioblastoma, and others using Flex-NK bispecific antibodies such as CYT-103 and CYT-303 + CYT-150 for hepatocellular carcinoma (Table 2). HebeCell’s technology platform is based on a proprietary method to generate large quantities of high-quality hiPSCs that can be differentiated into various cell types, including NK cells. The company employs a fully “feeder-free,” “serum-free,” and “xeno-free” differentiation process, aimed at ensuring product consistency and regulatory compliance. At present, HebeCell’s pipeline is limited to the preclinical stage, with ongoing studies evaluating the safety and efficacy of its CAR-NK cells in different tumor models, including solid cancers (Table 2). Shoreline Biosciences has developed a platform for the efficient production of NK cells and macrophages from hiPSCs, with potential implications in the treatment of various diseases, including cancer. A key innovation of this platform is the use of CRISPR-Cas9 gene editing to eliminate the CISH gene in hiPSC-derived NK cells. In a 2022 study, Bernareggi et al. demonstrated that NK cells generated using Shoreline platform exhibited strong cytotoxic activity against a range of cancer cell types in vitro. Moreover, these cells were well tolerated in a mouse model, showing no evidence of toxicity or adverse effects [102].

Overall, to date, clinical data support the safety of hiPSC-derived NK cell therapies and indicate encouraging preliminary signals of efficacy in hematologic malignancies. Nevertheless, the definitive clinical efficacy of hiPSC-derived NK cells has yet to be established in Phase II clinical trials, and initiation of Phase I trials for solid tumors is expected. The growing number of biotechnology companies focusing on the development of hiPSC-based cell therapies demonstrates the enormous market interest in this innovative approach [103].

**Table 2:** Landscape of companies developing hiPSC-derived cell therapies

Companies	Cell type	Target	Product Name	Therapeutic and engineering strategies	Therapeutic Area	Preclinical vs. Clinical	NCT ID	Status	Ref
**Allife Medical Science**	iPSC-NK	CD19	CD19 iCAR-NK	CAR.CD19 (full construct not disclosed)	R/R B-Cell Lymphoma	Phase I	NCT03824951	Unknown	-
**Allogene Therapeutics/Notch Therapeutics**	iPSC-T	-	AlloCAR T™	Not Disclosed	Not Disclosed	Preclinical	-	-	-
**BrightPath Biotherapeutics**	iPSC-NKT	-	BP2201	Unmodified	R/R HNSCC	Phase I	-	Not Listed in ClinicalTrials.gov	-
	iPSC-NKT	BCMA	BP2202	CAR.BCMA (full construct not disclosed)	MM	Preclinical	-	-	-
	iPSC-NKT	HER2	Not Disclosed	CAR.HER2 (full construct not disclosed)	OC	Preclinical	-	-	-
	iPSC-NKT	GD2	Not Disclosed	Unmodified + Anti-GD2 Monoclonal Antibody	Neuroblastoma	Preclinical	-	-	[104]
**Bristol Myers Squibb/Century Therapeutics**	iPSC-NK/T	-	CNTY-104	Not Disclosed	AML	Preclinical	-	-	-
	iPSC-NK/T	-	CNTY-106	Not Disclosed	MM	Preclinical	-	-	-
**Caribou Biosciences**	iPSC-NK	-	CB-020	CAR (full construct not disclosed); and further modifications not disclosed	Solid Tumors	Preclinical	-	-	-
**Cytovia Therapeutics/Cellectis/CytoLynx Therapeutics/Cytoimmune**	iPSC-NK	GPC3	CYT-503	CAR.GPC3 (full construct not disclosed)	HCC	Preclinical	-	-	-
	iPSC-NK	CD38	CYT-538	CAR.CD38 (full construct not disclosed)	MM	Preclinical	-	-	-
	iPSC-NK	EGFRvIII and EGFRwt	CYT-501	CAR.EGFRvIII (full construct not disclosed)	GBM	Preclinical	-	-	-
	iPSC-NK	GPC3	CYT-103	GPC3 Flex-NK™ Bispecific Antibodies Pre-complexed with iPSC-NK cells	HCC	Preclinical	-	-	-
	iPSC-NK	GPC3	CYT-303 + CYT150	GPC3 Flex-NK™ Bispecific Antibodies + Edited iPSC-NK cells; TGFβR2-/- (KO); mbIL-15 +/+ (KI)	HCC	Preclinical	-	-	[105–107]
**Century Therapeutics**	iPSC-NK	CD19	CNTY-101	CAR.CD19; soluble IL-15 (KI); EGFR safety switch (KI); HLA-I (KO); HLA-E (KI)	B-Cell Malignancies	Phase I (NHL; R/R CD19 + B-Cell Malignancies)	NCT05336409	Active, not recruiting	[108, 109]
	iPSC-γδ T	CD19, CD22	CNTY-102	CAR.CD19/22; IL-15RF (KI)	B-Cell Malignancies	Preclinical	-	-	[110]
	iPSC-γδ T	Nectin-4	CNTY-107	CAR.Nectin-4 (full construct not disclosed)	Solid Tumors	Preclinical	-	-	[111]
**CytoMed Therapeutics**	iPSC-γδ NKT	-	gdNKT Therapy	Not Disclosed	Solid Tumors	Preclinical	-	-	-
**Edigene/Neukio Biotherapeutics**	iPSC-NK	-	Not Disclosed	CAR (full construct not disclosed)	Not Disclosed	Preclinical	-	-	-
**Editas Medicine**	iPSC-NK	-	EDIT-202	CISH-/- TGFβR2-/- (KO); CD16 +/+ mbIL-15 +/+ (KI)	Solid Tumors	Preclinical	-	-	[112]
**Exacis Biotherapeutics**	iPSC-NK/T	-	ExaCELLS™	Not Disclosed	Not Disclosed	Preclinical	-	-	-
**Fate Therapeutics**	iPSC-NK/T	CD19	FT819	CAR.CD19-CD28-CD3ζ; TCR (KO)	Hematological Tumors	Phase I (B Cell Lymphoma; CLL; B-ALL)	NCT04629729	Active, not recruiting	[113]
	iPSC-T	HER2	FT825/Ono	hnCD16 (KI); IL7RF (KI); CD38 (KO); CAR.HER2; Synth CXCR2 (KI); Synth TGFβ (KI); TCR (KO) ± Monoclonal Antibodies	Solid Tumors	Phase I (Advanced Solid Tumor)	NCT06241456	Recruiting	[114]
	iPSC-NK	Multi-antigen	FT500	Unmodified ± Pembrolizumab (anti-PD1); ± Avelumab (anti-PDL1); ± Nivolumab (anti-PD1)	Solid Tumors	Phase I (Advanced Solid Tumor)	NCT03841110	Terminated	[115]
					Solid Tumors	Phase I (Advanced Solid Tumor)	NCT04106167	Terminated Observational Study	
	iPSC-NK	Multi-antigen	FT516	hnCD16 (KI); ± Rituximab (anti-CD20) or ± Obinutuzumab (anti-CD20) (NCT04023071); ± Avelumab (anti-PDL1) (NCT04551885); ± Enoblituzumab (anti-B7-H3) (NCT04630769)	Solid/Hematological Tumors	Phase I (AML; B Cell Lymphoma)	NCT04023071	Completed Updated Clinical Data (2025): ORR (all responses combined): 58% (32/55) CR: 44% (24/55) PR: 15% (8/55) Recommended phase 2 dose: 9 × 10⁸ cells/dose, 3 doses per 28-day cycle No dose-limiting toxicities reported Safety Highlights: Cytokine release syndrome: 2%, Grade 1 No neurotoxicity reported Grade ≥ 3 adverse events: Neutropenia: 84% Thrombocytopenia: 36% Anemia: 27% No treatment-related deaths	[100]
						Phase I (Solid Tumors)	NCT04551885	Terminated	-
						Phase I (OC; FTC; PPC)	NCT04630769	Completed Updated Clinical Data (2023): DLT: 0% (0/3) ORR: 0% (0/3) PFS (6 months): 33% (1/3) PFS (12 months): 0% (0/3) OS: 0% (0/3) CR: 0% (0/3) Safety Highlights: Anemia: 100% Anorexia 100% Thromboembolic event 33% Infections 33% No treatment-related deaths	-
	iPSC-NK	CD19, CD20	FT522	hnCD16 (KI); IL15RF (KI); CD38 (KO); CAR.CD19; Synth ADR (KI) ± Rituximab	B-cell Lymphoma	Phase I (R/R B Cell Lymphoma)	NCT05950334	Recruiting	[116]
	iPSC-NK	MICA/B, Multi-antigen	FT536	hnCD16 (KI); IL15RF (KI); CD38 (KO); CAR.MICA/B-NKG2D-2B4-CD3ζ; ± Avelumab or ± Pembrolizumab (anti-PD-1) or ± Nivolumab (anti-PD-1) or ± Atezolizumab (anti-PD-L1) or ± Trastuzumab (anti-HER2) or ± Cetuximab (anti-EGFR) or ± Amivantamab (anti-EGFR and MET) (NCT05395052)	Solid Tumors	Phase I (Solid Tumors)	NCT05395052	Terminated	[117]
						Phase I (GC; OC; FTC; PPC;)	NCT06342986	Recruiting	
	iPSC-NK	Multi-antigen	FT538	hnCD16 (KI); IL15RF (KI); CD38 (KO); ± Daratumumab (anti-CD38) or ± Elotuzumab (anti-SLAMF7) (NCT04614636); ± Daratumumab/rHuPH20 (NCT04714372); ± Enoblituzumab (NCT05708924)	Solid/Hematological Tumors	Phase I (MM; AML)	NCT04614636	Terminated	[118]
						Phase I (Solid Tumors)	NCT05069935	Terminated	
						Phase I (AML; MM; Monocytic Leukemia)	NCT04714372	Completed Updated Clinical Data (2024): DLT: 0% (0/9) ORR (12 months): 22,2% (2/9 of dose 4) PFS (12 months): 33% (3/9) OS (12 months): 33% (3/9) CR: 22,2% (2/9 of dose 4) Safety Highlights: Anemia: 55% Anorexia 44% Thromboembolic event 11% Infections 11% Heart failure 11% No treatment-related deaths	
						Phase I (OC; FTC; PPC)	NCT05708924	Terminated Updated Clinical Data (2025): The trial was terminated before the outcome measure data were collected.	
	iPSC-NK	B7H3, CD38	FT573	hnCD16 (KI); IL15RF (KI); CD38 (KO); CAR.B7H3; ± Daratumumab	Solid Tumors	Preclinical	-	-	[119]
	iPSC-NK	BCMA, CD38	FT576	hnCD16 (KI); IL15RF (KI); CD38 (KO); CAR.BCMA; ± Daratumumab	MM	Phase I (MM)	NCT05182073	Active, not recruiting	[120]
	iPSC-NK	CD19, CD20	FT596	hnCD16 (KI); IL15RF (KI); CAR.CD19-NKG2D-2B4-CD3ζ; ± Rituximab or Obinutuzumab (NCT04245722); ± Rituximab (NCT04555811); ± Rituximab and/or ± R-CHOP (NCT05934097)	Lymphoma/Leukemia	Phase I (B Cell Lymphoma)	NCT04245722	Terminated Regimen A: FT596 Monotherapy (*n* = 18) Regimen B: FT596 + Rituximab (*n* = 68) Updated Clinical Data (2025): ORR (all responses combined): 54% (Regimen B; all histologies) CR: 37% PR: 18% No dose-limiting toxicities in Regimen A; one DLT in Regimen B (prolonged grade 4 thrombocytopenia) Maximum tolerated dose not reached Recommended phase 2 dose: 1.8 × 10⁹ cells/dose, 3 doses per 28-day cycle (Regimen B) Safety Highlights: Low-grade cytokine release syndrome: Regimen A: 6%, Grade 1 Regimen B: 13%, Grades 1–2 No neurotoxicity reported Grade ≥ 3 adverse events: Neutropenia: Regimen A: 78%, Regimen B: 88% Thrombocytopenia: Regimen A: 39%, Regimen B: 49% Anemia: Regimen A: 39%, Regimen B: 44% No treatment-related deaths	[99]
						Phase I (NHL; DLBCL; HGBCL)	NCT04555811	Completed Updated Clinical Data (2024): DLT: 0% (0/7) R/P (12 months): 42% (3/7) PFS (12 months): 57% (4/7) Safety Highlights: Anemia: 28% Infections 14% No treatment-related deaths	-
						Phase I (B Cell Lymphoma)	NCT05934097	Withdrawn (Sponsor decision)	-
**Healios**	iPSC-NK	-	HLCN061	Not Disclosed	Solid Tumors	Preclinical	-	-	[121]
**Hebecell/Jacobio Pharmaceuticals**	iPSC-NK	-	HC101	Not Disclosed	Multiple Tumors	Preclinical	-	-	[122]
	iPSC-NK	-	HC102	Not Disclosed	MOC	Preclinical	-	-	
	iPSC-NK	-	HC103	Not Disclosed	Hematological Tumors	Preclinical	-	-	
	iPSC-NK	-	HC104	Not Disclosed	Gastric/Lung Cancer	Preclinical	-	-	
	iPSC-NK	-	HC105	Not Disclosed	Brain Tumors	Preclinical	-	-	
	iPSC-NK	-	HC106	Not Disclosed	NK-Resistant Tumors	Preclinical	-	-	
	iPSC-NK	-	HC111	Not Disclosed	Hematological Tumors	Preclinical	-	-	
	iPSC-NK	-	HC112	Not Disclosed	Colorectal/OC	Preclinical	-	-	
	iPSC-NK	-	HC121D	Not Disclosed	AML	Preclinical	-	-	
	iPSC-NK	-	HC122D	Not Disclosed	Solid Tumors	Preclinical	-	-	
**ONK Therapeutics**	iPSC-NK	-	ONKT106	CISH-/- (KO)	Not Disclosed	Preclinical	-	-	[123]
**PersonGen BioTherapeutics**	iPSC-NK	-	Not Disclosed	CAR	Not Disclosed	Preclinical	-	-	-
**SCG Cell Therapy**	Not Disclosed	-	SCG452	Not Disclosed	Solid Tumors	Preclinical	-	-	-
**Shoreline Biosciences**	iPSC-NK	-	Not Disclosed	CISH-/- (KO)	Not Disclosed	Preclinical	-	-	[124]
	iPSC-MACs	-	Not Disclosed	Not Disclosed	Not Disclosed	Preclinical	-	-	-
**Sorrento Therapeutics/Karolinska Institutet**	iPSC-NK	-	Not Disclosed	Not Disclosed	Not Disclosed	Preclinical	-	-	-
**Thyas Co. Ltd.**	iPSC-NK	GPC3	Not Disclosed	CAR	Solid Tumors	Phase I	-	Not Listed in ClinicalTrials.gov	[125]
	iPSC-T	-	Not Disclosed	CAR	Solid Tumors	Preclinical	-	-	-
	iPSC-T	-	Not Disclosed	TCR	Solid Tumors	Preclinical	-	-	-
**Zhejiang University**	iPSC-NK	CLL1 or CD33	Not Disclosed	CAR.CLL1 or CAR.CD33 (full construct not disclosed)	AML	Phase I (AML)	NCT06367673	Recruiting	-
	iPSC-NK	CLL1	Not Disclosed	CAR.CLL1 (full construct not disclosed)	AML	Phase I (AML)	NCT06027853	Recruiting	-
**Xyphos Biosciences**	Not Disclosed	-	Not Disclosed	Not Disclosed	Not Disclosed	Preclinical	-	-	-

*Actual or Estimated

*Abbreviations*: *iPSC* Induced pluripotent stem cells, *NK* Natural Killer, *CAR* Chimeric Antigen Receptor, *NKT* Natural Killer T cells, *R/R* Relapsed or refractory, *HNSCC* Head and Neck Squamous Cell Carcinoma, *BCMA* B-Cell Maturation Antigen, *MM* Multiple Myeloma, *HER2* Human Epidermal growth factor Receptor 2, *OC* Ovarian Adenocarcinoma, *AML* Acute Myeloid Leukimia, *GPC3* Glypican-3, *KI* Knock-In, *KO* Knock-out, *HCC* Hepatocellular Carcinoma, *EGFR* Epidermal Growth Factor Receptor, *GBM* Glioblastoma Multiforme, *HLA* Human Leukocyte Antigen, *RF* Receptor Fusion protein, *gdNKT* Gamma-delta Natural Killer T cells, *CISH* Cytokine-Inducible SH2-containing protein, *TGFβ* Transforming Growth Factor, *CXCR2* C-X-C Chemokine Receptor type 2, *hnCD16* high-affinity non-cleavable CD16, *mbIL-15* Membrane-bound Interleukin-15, *CLL* Chronic Lymphocytic Leukemia, *B-ALL* Precursor B-Cell Acute Lymphoblastic Leukemia, *TCR* T Cell Receptor, *OC* Ovarian Cancer, *FTC* Fallopian Tube Carcinoma, *PPC* Primary Peritoneal Carcinoma, *ADR* Alloimmune Defense Receptor, *ORR* Objective Response Rate, *CR* Complete Response, *PR* Partial Response, *DLT* Dose Limiting Toxicity, *PFS* Progression Free Survival, *OS* Overall Survival, *R/P* Relapse/Progression, *MICA/B* MHC class I polypeptide–related sequence A and B, *MET* Mesenchymal–Epithelial Transition factor, *SLAMF7* Signaling Lymphocytic Activation Molecule Family member 7, *rHuPH20* recombinant human hyaluronidase PH20, *GC* Gynecologic Cancer, *PPC* Primary Peritoneal Cavity Cancer, *DLBCL* Diffuse Large B-Cell Lymphoma, *HGBCL* High-Grade B-Cell Lymphoma, *MOC* Metastatic Ovarian Cancer, *MACs* Macrophages, *CLL1* C-type lectin-like molecule-1

## Challenges

Despite the promise of hiPSC-derived NK cell therapy to address the limitations of primary NK cell sources, and the encouraging results emerging from preclinical and early clinical studies, no NK cell therapy has yet received FDA approval, unlike several T cell-based therapies, such as CAR T cells. This gap underscores that allogeneic NK cell therapies, including those derived from hiPSCs, continue to face key challenges that must be resolved before regulatory approval can be achieved. These challenges include safety, efficacy, immune compatibility, and large-scale manufacturing. In the following sections, we discuss these hurdles and highlight current strategies under investigation to overcome them.

### Addressing safety and efficacy

The genetic and epigenetic stability of adoptive cell therapies is increasingly recognized as a critical factor for ensuring long-term safety. A recent alert from FDA regarding approved CAR-T cell therapies has raised concerns about serious genomic risks after the report of at least 3 T cell malignancies developing after treatment, where the malignant cells were found to express the CAR construct, indicating a potential transformation caused by the therapy itself [126, 127]. Although these events are rare, their detection has led to a boxed warning for all therapies with genetically engineered cells. To date, there have been no reported cases of malignant transformation in NK cell-based therapies. However, since the application of hiPSC-derived NK cell therapies may involve multiple genetic engineering steps, the potential for insertional mutagenesis, epigenetic dysregulation, or the selection of pre-malignant clones remains a significant concern, particularly as CAR-based therapies are being investigated in earlier disease stages, autoimmune conditions, and infectious diseases [128–130]. Thus, the importance of thoroughly evaluating the genetic and epigenetic consequences of ex vivo manipulation, genomic editing, viral vector-mediated gene transfer, and clonal expansion is also relevant to NK cell manufacturing from hiPSC.

In the context of genetic stability, NK cells derived from hiPSCs present both advantages and challenges. The production of hiPSC-derived NK cells typically requires prolonged in vitro processing, including reprogramming, genetic engineering, and differentiation, which may increase the risk of acquiring genetic abnormalities. Furthermore, the generation of hiPSCs through cellular reprogramming is frequently accompanied by genomic instability. Several analyses have reported an average of 5–6 non-synonymous coding mutations per hiPSC line, including alterations in genes linked to tumorigenesis [131–134]. These mutations are not randomly distributed, but tend to cluster in genomic regions particularly susceptible during reprogramming, suggesting non-stochastic mutational mechanisms [135]. An analysis of several hundred hiPSC lines revealed substantial variability in point mutations, aneuploidies, and structural alterations, influenced by both the genetic background of the donor and the source cell type. In addition, as stressed above, hiPSCs often retain epigenetic marks from their tissue of origin, which can limit differentiation potential. A recent strategy based on chromatin modulation has proven effective in erasing this residual epigenetic memory and restoring a more embryonic-like epigenetic profile.

However, utilizing hiPSCs offers a significant advantage by facilitating early and thorough genetic screening prior to clinical application. By selecting a master hiPSC line free from oncogenic mutations, chromosomal abnormalities, or non-specific genome editing, the risk of genetic instability in the final cell product can be minimized. Additionally, hiPSCs can be engineered with safety switches, such as inducible caspase 9 [136] or herpes simplex virus thymidine kinase [137], which can trigger apoptosis if toxicity or malignant transformation occurs. Together, these strategies provide critical safeguards to enhance the consistency and safety of hiPSC-derived NK cell therapies.

A key technical challenge in hiPSC genetic engineering is the risk of transgene silencing during differentiation. Viral promoters such as CMV are particularly prone to epigenetic inactivation in hiPSCs, often resulting in reduced or even complete loss of expression [131–134]. By contrast, the EF1α promoter is widely used for its transcriptional stability, while the CAG hybrid promoter, though less commonly employed, has also shown improved resistance to silencing [138, 139].

Stable expression also depends on the genomic insertion site. “Safe harbors” loci, such as AAVS1, hROSA26, and CCR5, offer a permissive chromatin environment that supports sustained transcription while minimizing disruption of essential genes or oncogenic activation. Targeted integration into AAVS1, for example, maintains stable transgene expression in both pluripotent and differentiated states, reducing epigenetic silencing and preserving genomic integrity [140]. Despite these challenges, engineering hiPSCs rather than their differentiated progeny offers clear advantages in terms of cost, product standardization, and the possibility of introducing multiple modifications to enhance performance. By contrast, post-differentiation modification is less efficient, more heterogeneous, and largely defeats the rationale for using hiPSCs in the first place; in such settings, alternative primary sources may represent a more suitable option.

A major challenge limiting the long-term efficacy of allogeneic NK cell-based immunotherapies is their short half-life and limited persistence after infusion [141, 142]. Although this short lifespan can contribute to a favorable safety profile, including a low incidence of CRS and neurotoxicity [7], it reduces the ability to clear tumors over time and limits durable clinical responses. To address this, lymphodepleting regimens before allogeneic NK cell infusion help reduce immune rejection and improve the effectiveness of allogeneic CAR-NK therapies [143]. In addition, cytokine-based strategies have also been developed to improve allogeneic NK cell survival, help induce memory-like properties, and enhance their response to repeated antigen exposure [144] promote NK cell survival, and among these, IL-15 has been particularly effective in clinical applications, supporting NK cell expansion and activation up to over a year after infusion, while maintaining a low toxicity profile [145–147]. Another strategy to sustain therapeutic effects over time involves administering multiple infusions of NK cells. Preliminary data from four Phase I clinical trials involving patients with solid tumors [148] and hematological malignancies [99, 149, 150] suggest that repeated CAR-NK cell infusions are well-tolerated and do not result in serious adverse events. However, these findings are based on interim results from a limited cohort of just 31 patients. Therefore, additional studies are necessary to more conclusively evaluate the efficacy and long-term safety of this therapeutic approach.

### Current regulatory framework for hiPSC-NK therapy

The smooth and rapid clinical translation of cellular therapeutics, such as hiPSC-derived NK cells, depends on the existence of a standard and clearly defined regulatory framework. In the case of hiPSC-based therapies, the regulatory frameworks differ between the European Union (EU) and the United States (US), reflecting regional priorities in balancing innovation, safety, and access.

In the EU, hiPSC-derived products fall under the regulatory framework for ATMPs, which are evaluated through a centralized approval process coordinated by the European Medicines Agency (EMA) under Regulation (EC) No. 1394/2007 and Directive 2001/83/EC (26). In the US, the FDA regulates hiPSC-based therapies as biologics or Human Cells, Tissues, and Cellular and Tissue-Based Products (HCT/Ps), under 21 Code of Federal Regulations (CFR) Part 1271 and the Public Health Service Act (PHSA) [151]. However, both agencies provide guidance in quality standards for commercial development, guidance for early-phase trials is less clearly defined, complicating the path to first-in-human studies.

Moreover, while both agencies aim to ensure the safety and efficacy of hiPSC-based therapies, their approaches differ. The EMA enforces comprehensive GMP standards, mandates environmental risk assessments, and requires the involvement of separate personnel for production and quality control even in early phases. In contrast, the FDA allows more flexibility in early-stage trials, requiring full GMP compliance only in later phases, including fewer environmental controls and permitting a single individual to oversee both production and quality control. Despite collaborative efforts to achieve regulatory harmonization, challenges still remain, since differences in national laws, policy update timelines, and post-marketing surveillance models continue to complicate full alignment. In order to complement existing legal frameworks and inform the interpretation and development of laws applicable to stem cell research, the ISSCR (International Society for Stem Cell Research) has published the Guidelines for Stem Cell Research and Clinical Translation [152]. Joint regulatory guidance on hiPSC-specific issues, including genetic stability, tumorigenicity, and long-term safety, has led to more consistent approaches to testing. However, in some cases, standards on assay requirements and acceptable results are still unclear. For example, although current regulatory guidelines still accept karyotyping as adequate to test genomic stability, the increasing complexity of gene-editing strategies in hiPSC-derived therapies may necessitate more comprehensive genomic analyses, such as off-target analysis and whole-genome sequencing, which would require the establishment of new standards to evaluate the results. Both EMA and FDA recognize genomic stability testing as a critical safety requirement for iPSC–derived ATMPs. However, EMA generally adopts a more precautionary stance, with explicit regulations requiring comprehensive cytogenetic and molecular analyses (G-banding, FISH, array CGH, SNP array, or NGS-based methods) at the MCB, working cell bank (WCB), and final product stages, with low tolerance for subclonal abnormalities. In contrast, FDA guidance adopts a more flexible, case-by-case approach, allowing sponsors to justify fit-for-purpose methods in early clinical development.

Although to date, no hiPSC-derived therapy has a published full marketing-authorization ATMP dossier, several hiPSC-derived programmes have publicly accessible regulatory milestones (IND clearances, CTN/CTA notifications, and clinical-trial registrations) that provide practical examples of regulatory submissions for such products.

### Cost considerations

In developing cell-based therapies, in addition to meeting regulatory standards and ensuring thorough product characterization, developers must carefully evaluate manufacturing models and their economic implications. Allogeneic cellular therapies, particularly those derived from hiPSCs, offer the potential to create “off-the-shelf” products that overcome key limitations of autologous therapies, namely high costs, limited accessibility, and inconsistent product quality, which restrict their ability to meet growing global demand, especially in developing countries. The development of hiPSC-derived NK therapies is theoretically more economically viable because it eliminates costly individualized services, such as patient-specific cell banking, customized processing, and repeated quality control testing. Instead, it relies on standardized production, which is expected to reduce the cost per treatment dose over time as economies of scale are achieved [153]. Nevertheless, translating hiPSC-derived products into clinical applications presents both technical and financial challenges. Currently, the high cost of generating hiPSCs and differentiating them into therapeutic NK cells restricts their application to severe or life-threatening conditions with no effective treatment options. However, as technological expertise grows, supply chains mature, and regulatory processes become more efficient, both production costs and associated risks are expected to decline. For instance, data from autologous CAR T cell production show that labor alone accounts for 20–32% of total manufacturing costs. Material costs make up about 15–19%, while equipment represents just 2–4% [154]. This underscores the critical need to reduce labor dependency, particularly through automation. Automation is increasingly viewed as a key enabler of scalability and efficiency [155]. Furthermore, integrating digital platforms into quality assurance processes can improve operational efficiency and ease of system maintenance [156]. One study demonstrated that manual hiPSC production is 42% more expensive than automated processes, largely due to personnel costs, which dropped from €4 million to €630,000 over eight years with automation [157]. Despite these advantages, automation in stem cell manufacturing also presents unique challenges: cells may respond unpredictably to artificial surfaces, and bioreactor systems can display altered mass transfer dynamics. Automated, closed bioreactor systems have already demonstrated success in producing CAR T cells at a clinical scale [158, 159]. While automated, closed bioreactor systems have shown promise in manufacturing NK cell therapies [160, 161], their application to NK cell production from stem cells remains largely unexplored.

Simultaneously, innovations in reagents and biomaterials are contributing to both cost reduction and improved therapeutic outcomes. These advances include more efficient reprogramming agents, optimized media formulations that promote faster cell growth while minimizing undesired differentiation, and robust culture substrates. Taken together, the shift toward allogeneic, hiPSC-derived NK therapies supported by advances in automation, optimized media formulations, and innovative biomaterials, represents a critical step toward scalable, cost-effective, and globally accessible cell-based treatments.

## Concluding remarks

Allogeneic hiPSC-derived NK cell-based therapies have emerged as a promising “off-the-shelf” immunotherapy for patients with advanced cancers, offering a favorable safety profile and potential clinical benefit. To date, available clinical data confirm their safety and provide preliminary evidence of efficacy in hematologic malignancies; however, definitive benefit remains to be demonstrated in Phase II trials, and first-in-human studies in solid tumors are only now being initiated. hiPSC-derived NK cell therapy has the potential to provide solutions to cost, accessibility, and quality issues of autologous adoptive cell therapy. The hiPSC platform, in combination with advances in genome editing technologies, can accommodate a series of novel genetic engineering approaches aimed at tailoring hiPSC-NK cells to specific tumor microenvironments and optimize their persistence and overall anti-tumor efficacy. This field is rapidly evolving, with significant investment from biotechnology companies that recognize the potential of hiPSCs as a scalable source of NK cells that can be propagated and differentiated in vitro in large quantities. As this field progresses, the path to clinical application and regulatory approval requires addressing challenges related to product safety and quality assurance, the harmonization and standardization of regulatory guidelines, and the refinement of scalable and automated manufacturing processes. Continued investment will be essential to overcome current barriers and unlock the full therapeutic potential of hiPSC-NK cell therapy across diverse clinical applications.

## Data Availability

All data and materials are included in the references.

## Abbreviations

aAPCs: Artificial Antigen-Presenting Cells
ACT: Adoptive Cell Therapies
ADAM17: A Disintegrin And Metalloprotease-17
ADCC: Antibody-Dependent Cellular Cytotoxicity
ASM: Acid Sphingomyelinase
ATMPs: Advanced Therapy Medicinal Products
BLA: Biologics License Application
BM: Bone Marrow
CAGR: Compound Annual Growth Rate
CAR: Chimeric Antigen Receptor
CB: Cord Blood
CIITA: Class II Major Histocompatibility Complex Transactivator
CISH: Cytokine inducible SH2-containing protein
CLPs: Common Lymphoid Progenitors
CMPs: Common Myeloid Progenitors
CRISPR: Clustered Regularly Interspaced Short Palindromic Repeats
CRS: Cytokine Release Syndrome
CTLA-4: Cytotoxic T-Lymphocyte-Associated Protein 4
EMA: European Medicines Agency
FDA: Food and Drug Administration
GMP: Good Manufacturing Practices
GvHD: Graft-versus-Host Disease
HCMV: Human Cytomegalovirus
hiPSC: Human Induced Pluripotent Stem Cell
HLA: Human Leukocyte Antigens
hnCD16: High-affinity, non-cleavable CD16a
HCT/Ps: Human cells, tissues, and cellular and tissue-based products
ICH: International Council for Harmonization
IND: Investigational New Drug
ITAMs: Immuno-tyrosine activation motifs
ITSM: Immunoreceptor Tyrosine-based Switch Motif
Klf4: Krüppel-like factor 4
LAG-3: Lymphocyte-Activation Gene 3
MACs: Macrophages
MCB: Master Cell Banks
NK: Natural Killer
NKT: Natural Killer T
NSMASE: Neutral Sphingomyelinases
Oct3/4: Octamer-binding transcription factor 3/4
PB: Peripheral Blood
PD1: Programmed cell Death Protein 1
PDL1: Programmed Death-Ligand 1
PHSA: Public Health Service Act
PSA: Parallel Scientific Advice
scFv: Single-chain variable Fragment
SOCS: Suppressor of Cytokine Signaling
Sox2: SRY (Sex Determining Region Y)-box 2
TGF-β: Transforming Growth Factor beta
TGFβR2: Transforming Growth Factor, beta receptor II
TIGIT: T cell Immunoreceptor with Ig and ITIM domains
TIM-3: T-cell Immunoglobulin and Mucin-domain containing-3
TME: Tumor Microenvironment
TRAILv: TNF-Related Apoptosis-Inducing Ligand variant
c-Myc: Cellular-myelocytomatosis
